# Pelvic-Floor Dysfunction Prevention in Prepartum and Postpartum Periods

**DOI:** 10.3390/medicina57040387

**Published:** 2021-04-16

**Authors:** Karolina Eva Romeikienė, Daiva Bartkevičienė

**Affiliations:** Clinic of Obstetrics and Gynecology, Faculty of Medicine, Institute of Clinical Medicine, Vilnius University, LT-01513 Vilnius, Lithuania; eva.romeikiene@gmail.com

**Keywords:** pelvic-floor rehabilitation prepartum, pelvic-floor rehabilitation postpartum, pelvic-floor muscle training, pelvic organ prolapse prevention, pelvic-floor dysfunction prevention

## Abstract

Every woman needs to know about the importance of the function of pelvic-floor muscles and pelvic organ prolapse prevention, especially pregnant women because parity and labor are the factors which have the biggest influence on having pelvic organ prolapse in the future. In this article, we searched for methods of training and rehabilitation in prepartum and postpartum periods and their effectiveness. The search for publications in English was made in two databases during the period from August 2020 to October 2020 in Cochrane Library and PubMed. 77 articles were left in total after selection—9 systematic reviews and 68 clinical trials. Existing full-text papers were reviewed after this selection. Unfinished randomized clinical trials, those which were designed as strategies for national health systems, and those which were not pelvic-floor muscle-training-specified were excluded after this step. Most trials were high to moderate overall risk of bias. Many of reviews had low quality of evidence. Despite clinical heterogeneity among the clinical trials, pelvic-floor muscle training shows promising results. Most of the studies demonstrate the positive effect of pelvic-floor muscle training in prepartum and postpartum periods on pelvic-floor dysfunction prevention, in particular in urinary incontinence symptoms. However more high-quality, standardized, long-follow-up-period studies are needed.

## 1. Introduction

Pelvic organ prolapse (POP) affects about 50% of women undergoing routine gynecological examination [1]. It is a common condition among parous women and has negative effect on the quality of life in general and especially affecting sexual life and self-confidence. The amount of POP is likely to increase in the future. It is thought that in 2050 the number of women with POP in the USA will increase by about 46% [2].

Main POP risk factors are parity, advancing age, obesity, and others—race and ethnicity, collagen abnormalities, hysterectomy, elevated intraabdominal pressure, and family history [3,4,5,6,7,8,9,10]. Most of the POP risk factors are unchangeable, which emphasizes the role of POP prevention. Even though the incidence of POP increases with age, women at young age should start intervening to prevent this condition from happening in the future [3,5,9]. Every woman needs to know about the importance of the function of pelvic-floor muscles (PFM) and POP prevention, especially pregnant women since parity and labor are the factors which have the biggest influence on having POP in the future [3,4,9,11]. Compared to natural delivery, Cesarean delivery mode is not completely protective [12]. There are other health problems such as urinary incontinence (UI), anal incontinence (AI), or sexual disfunction which usually goes with POP and has similar risk factors and etiology. POP is one of the most common diagnoses composing pelvic-floor dysfunction (PFD). According to the International Urogynecological Association (IUGA) and the International Continence Society (ICS), who made a joint report on the terminology for Female Pelvic-Floor Dysfunction, PFD is a wider term covering the following diagnosis: POP, urodynamic stress incontinence (SUI), detrusor overactivity, bladder oversensitivity, voiding dysfunction, recurrent urinary tract infections, and also symptoms such as anal incontinence, dyspareunia, vaginal laxity, and perineal and pelvic pain [13]. Every pregnant woman should learn how to prevent pelvic-floor trauma during labor and how to rehabilitate PFM after labor. PFM training (PFMT) has promising results in POP and PFD prevention and even treatment in early stages of these conditions. Although there is a lack of long-term follow-up studies, existing clinical trials and consensus of experts shows compliance with the use of PFMT [14,15]. In some countries, there are pregnancy and post-partum-orientated pelvic-floor rehabilitation (PFR) programs which contain PFMT. According to the “International Survey Questionnaire on Pelvic-Floor Rehabilitation After Childbirth”, countries in Europe are much more likely to recommend and fund pelvic-floor rehabilitation programs after birth than USA or Asian countries [16]. In Lithuania, we do not have national programs of pelvic physical therapy for patients before and early after birth. In this review, we explored methods of training and rehabilitation in prepartum and postpartum periods and their effectiveness.

## 2. Search Methods

The search for publications in English was made in two databases during the period from August 2020 to October 2020 in Cochrane Library and PubMed. Keywords for the search were different combinations of the following phrases: “woman pelvic-floor rehabilitation” and “prolapse prevention after delivery”. The selected articles met the following criteria: prepartum, delivery, or postpartum-related (words: pregnancy, obstetric, antenatal, postnatal, delivery, prepartum, postpartum, primiparous, childbirth, obstetrical perineal tears were mentioned in the article title) and prevention-related (words: training, exercise, prevention, treatment, physiotherapy, pelvic-floor interventions, rehabilitation). The titles of selected articles, abstracts, and full-text articles were screened by two independent reviewers.

At the beginning, a keyword search was conducted in “Medical subject headings” (MeSH) tree, keywords “pelvic floor dysfunction” and “pelvic organs prolapse” were suggested, but no keywords related with pelvic-floor rehabilitation during or after pregnancy were found. Keywords such as “pelvic floor dysfunction prevention prepartum”; “pelvic floor rehabilitation postpartum”; were used while researching Cochrane Library and PubMed, but the search results were small numbers of publications and did not suit the desirable theme. The most promising results appeared after looking for the most suitable keywords for this research “woman pelvic floor rehabilitation”, “pelvic floor dysfunction prevention after delivery”.

By using “woman pelvic floor rehabilitation” in the Cochrane library database, 5 Cochrane reviews from the period from 2008 to 2018 were found but none of them were prepartum, delivery, or postpartum and prevention-related. 213 clinical trials from the period from 1991 to 2020 were found, of which only 30 were prepartum, delivery, or postpartum and prevention-related.

By using “pelvic floor dysfunction prevention after delivery” no reviews were found, only 39 clinical trials of which 17 met the above-mentioned criteria.

By using “woman pelvic floor rehabilitation” in the PubMed database and using a systematic review filter, 92 articles from the period from 1998 to 2020 were found. Only 9 of them met the criteria. There were 380 clinical trials found, using clinical trial filter from 1984 to 2020 period. 51 of them were prepartum, delivery or postpartum-related.

By using “pelvic floor dysfunction prevention after delivery” 4 systematic reviews were found, 3 of them were related to prolapse and delivery, but none of them were related to prevention. There were 10 clinical trials, of which 6 met the criteria.

**Figure 1:**
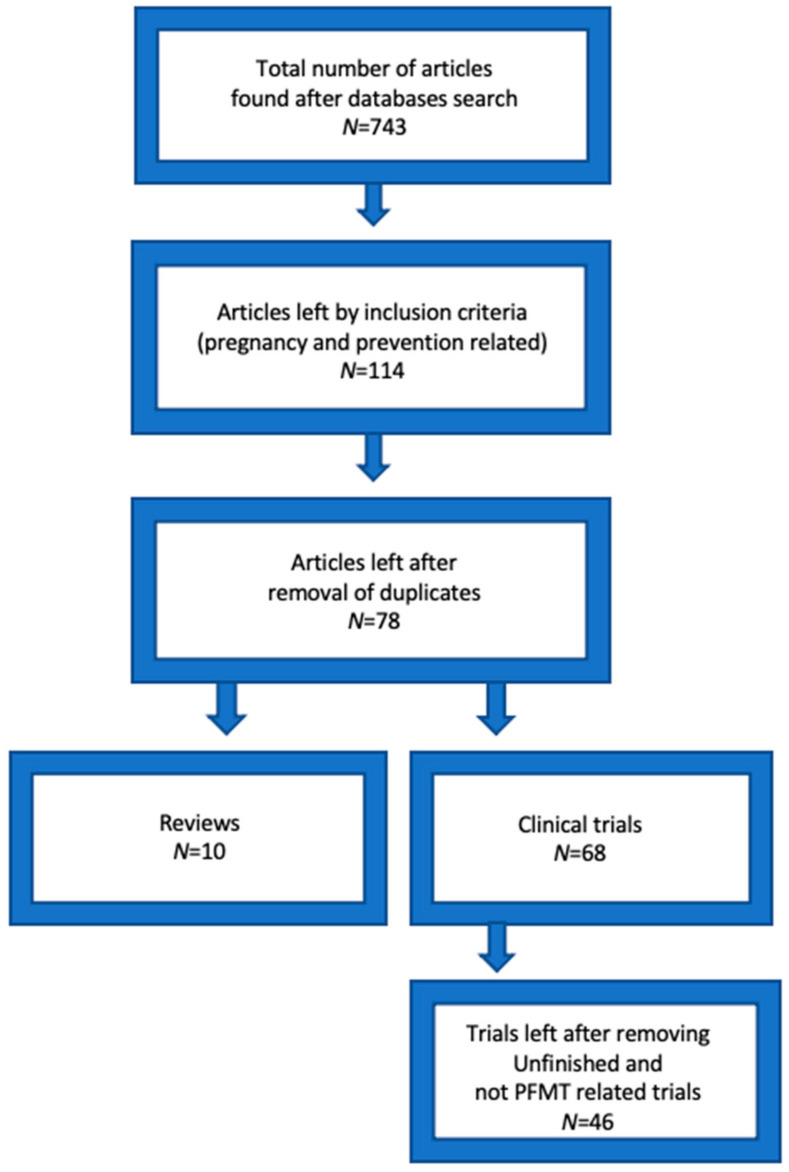
Studies selection flow.

After comparing selected articles from two databases and removing repeating articles, 77 articles were left in total as well as 9 systematic reviews and 68 clinical trials. Existing full-text papers were reviewed after this selection. Unfinished clinical trials, those which were designed as strategies for national health systems and those which were not PFMT-specific were excluded, for example: “general fitness classes in pregnancy effect on postpartum period” (Figure 1).

Most of the clinical trials were studies of intervention group (for example: PFMT supervised by specialist, PFMT using various rehabilitation devices) versus control group (for example: PFMT at home, no PFMT) in pregnant and postpartum women, most of them were analyzed by intention-to-treat principle. Randomized clinical trials (RCT) were grouped by the symptoms they treated (Table 1). For assessing risk of bias, RoB 2 tool was used in RCTs. Most of the trials were unclear risk of selection bias because of insufficient information provided on random sequence generation and high to moderate overall risk, due to low numbers of participants, participants, and personnel blinding errors, and short follow-up terms.

## 3. PFMT and Sexual Life Quality

PFMT effect on women’s sexual life was analyzed in 7 RCTs. All the RCTs analyzed the postpartum period. Only 4 RCTs used questionnaires or scales to evaluate sexual function (SF): Marinoff Dyspareunia Scale, female sexual index (FSFI), sexual self-efficacy questionnaire, International consultation on incontinence (ICIQ) modular questionnaire—vaginal symptoms (ICIQ-VS), and ICIQ sexual matters module ICIQ-FLUTSsex. There was a tendency of improvement in SF postpartum by doing PFMT or PFMT combined with intravaginal transcutaneous electrical nerve stimulation (TENS), there was improvement in vaginal laxity, arousal, lubrication, orgasm, and dyspareunia, no evidence of improvement found of using far-infrared radiation (FIR) device. There was an improvement in desire and pain symptoms in control group (no PFMT) within a 3-month period from the 4^th^ to 7^th^ month postpartum in one trial, but another trial showed improvement in pain symptoms with intensive PFMT, where both trials used FSFI questionnaires [17,18]. There were no major differences in vaginal symptoms between PFMT and control groups in one trial, but the PFMT group showed improvement in vaginal laxity symptoms, especially when there were levator ani muscle defects [17]. There is only low quality of evidence due to small sample sizes, short follow-up, randomization and blinding errors, and lack of standardized training reporting [19,20,21,22,23,24,25,26].

## 4. PFMT and Pelvic-Floor Dysfunction

A mix of symptoms—POP, UI, and AI—were analyzed in 10 RCTs. Most of the RCTs trained women during the postpartum period. There were PFMT used together with rehabilitation devices such as: Direct Vagina Low Voltage Low Frequency Electric Stimulation (DES), transvaginal electrical stimulation (TVES), EMG-triggered neuromuscular stimulation, electrical stimulation (ES) with biofeedback treatment, sacral neuromodulation, bipolar vaginal radiofrequency device (VotivaTM, InMode), EmbaGYN, Magic Kegel Master devices. Other treatment procedures included injections of collagen. In addition, there was classical PFMT versus control group RCTs. ES with biofeedback and DES showed promising results in decreasing POP symptoms. In one trial, a group of patients, whose POP and UI symptoms improved the most, and started PFMT very early second day postpartum, regardless of whether they had episiotomy or second-degree perineum laceration, they received DES therapy 6 weeks postpartum [25]. TVES showed no evidence of improvement in PFD questionnaires or muscle strength, but there was a higher rate of correct PFM contraction in the group with weak PFM, which received TVES 5 times in 7 to 14 weeks postpartum [27]. Sacral neuromodulation showed improvement in AI, UI, and life-quality symptoms. Vaginal radiofrequency devices showed no evidence of improvement in POP, UI, or AI symptoms. EmbaGYN and Magic Kegel Master devices showed significant improvement in UI symptoms. Three PFMT versus control group RCTs showed no significant improvement in any of symptoms. One RCT with a high sample size and long follow-up showed promising results in UI symptoms, but the effect did not last for 12 months (Glazener C M 2001 and 2017). PFMT together with rehabilitation devices may improve PFD symptoms, but the results should be evaluated with care, due to small samples, selective reporting, and selection and performance biases.

One RCT analyzed PFMT effect in postnatal AI treatment. There was a significant difference in the reduction of St. Mark’s scores in favor of PFMT.

One RCT analyzed the PFMT effect on prevention of POP postpartum, but there was no significant improvement [25,26,27,28,29,30,31,32,33,34,35,36].

## 5. PFMT and Obstetrical Injuries

Three RCTs analyzed various techniques to avoid obstetrical injury and/or episiotomy. Two RCTs analyzed antenatal use of Epi No device; neither found significantly lower incidence of anal sphincter, levator ani muscle injury or episiotomy in the Epi No group. One RCT compared antenatal perineal massage with PFMT to standard care. There was a significant reduction in episiotomy rates in the intervention group, also less third- to fourth-degree tears and less postpartum perineal pain. Two RCTs analyzed postpartum PFMT, one when there was an obstetrical anal sphincter injury (OASIS) and another when there was third-degree tears. None of them found statistically significant improvement after intervention [37,38,39,40,41].

## 6. PFMT and UI Prevention and Treatment

The highest number of RCTs—22—analyzed prepartum and/or postpartum PFMT effect on UI. To evaluate the effect of PFMT, six of them used specialized questionnaires: International Consultation on Incontinence Questionnaire Overactive Bladder (ICIQ-OAB), Urogenital Distress Inventory (UDI), Incontinence Impact Questionnaire (IIQ), International Consultation on Incontinence Questionnaire-short form (ICIQ-UI SF), International Consultation on Incontinence Questionnaire Lower Urinary Tract Symptoms Quality of Life Module (ICIQ-LUTSqol), and in most of them there was a statistically significant difference in favor of PFMT. Five used self-reported symptoms of UI. Two RCTs used Pad test to evaluate UI, one used bladder neck mobility. Most of the trials showed the positive effect of PFMT on UI, and PFMT group had less UI events in late pregnancy. One trial showed that written instructions of how to perform PFMT gives similar result as PFMT with specialist follow-up. Three trials showed the great effect of combined rehabilitation methods, e.g., PFMT with ES. Positive PFMT together with ES had long-lasting effect on UI; follow-up one year after intervention was conducted in one trial [42]. Preventative PFMT effect on UI was still present after 7 years in one trial [43]. One big sample (723 patients recruited, 234 included in trial) trial found that PFMT was non-effective in preventing future incontinence [44]. Only three RCTs followed up women after more than 6 months [42,43,44,45,46,47,48,49,50,51,52,53,54,55,56,57,58,59,60,61,62,63].

## 7. Systematic Reviews about Antenatal and Postnatal PFMT

Systematic review characteristics are shown in Table 2. For assessing quality of evidence, the GRADE tool was used in systematic reviews. Many of the reviews were downgraded to low or very low quality of evidence, due to small samples of RCTs, low evidence quality of RCTs, high heterogeneity, and selective reporting biases. The main systematic review, which is continuous and is regularly updated and provides the highest level of evidence is *Woodley et al*. in Cochrane Systematic Review. The main conclusions from the reviews were that there is a lack of high-quality randomized and standardized studies. It is very hard to avoid randomization bias in PFMT-based interventions, due to difficulties of blinding. Despite clinical heterogeneity among the RCTs, PFMT shows promising results in reducing UI and improving quality of life, SF, and AI scores after pregnancy [64,65,66,67,68,69,70,71,72].

## 8. Discussion

Most of the studies agree with the use of PFMT in PFD prevention in prepartum and postpartum periods, although more high-quality studies are needed.

Good results are demonstrated by using biofeedback therapy, which allows patients to see and evaluate their progress. Studies which have used biofeedback training also had longer follow-up period from 6 months to 7 years [26,43,53].

Higher-quality studies are needed to investigate SF. Higher sample sizes, randomization of participants, and at least 3 to 12 months of follow-up is needed. No results of SF in prepartum period are known, although this period is hard to evaluate because of physical and psychological changes in women. PFMT improves muscle mass and tone, which is the opposite for looseness or laxity. PFMT helps to reach targeted results if patients experience symptoms such as “vagina feels loose or lax” [21]. If patients experience dyspareunia symptoms, additional effects could be reached by adding TENS to the PFMT program [22]. Some of the symptoms may improve within the time after delivery without PFMT, but PFMT groups in most of the trials reached improvement in greater variety of symptoms. Also, PFMT effectively improved SF when there were muscle defects, and intensive PFMT may help to reduce pain during intercourse and painful perineal scar formation [17,18].

Higher-quality evidence is needed about rehabilitation device (ES, TVES, FIR, sacral neuromodulation, radiofrequency, Kegel trainers) usage to treat prepartum and postpartum PFD. ES may be useful to relieve pain and muscle hypertonus; in this way it may improve dyspareunia symptoms. ES and TVES may be not that effective in improving muscle mass and tonus but it may help to teach patients with extremely weak perineal muscles how to perform a correct PFM contraction [20,27].

Kegel training devices may improve PFD symptoms and increase muscle tone; it is comfortable for patients, because they can use the devices at home, but there is a lack of strong scientific evidence. Usage of the device should be precisely documented, which is hard when patients are training at home all by themselves, therefore training time and number and strength of contractions and other parameters important for device effect evaluation may be poorly documented and not suitable to compare between study participants [33]. The Epi No device does not significantly reduce perineal trauma and episiotomy rates. Perineal massage and PFMT may give promising results in reducing perineal tears and episiotomy rates, but more high-quality studies with well documented technique and study protocols are needed to evaluate perineal muscle relaxation techniques’ additional effects in avoiding perineal trauma [37,38,39,40,41].

There are established results that antenatal PFMT helps to prevent UI in late pregnancy and reduce UI rates after delivery. Preventative PFMT effect is long-lasting. The best results are in continent women when they start structured PFMT at early pregnancy. The highest numbers of studies evaluate this symptom and here we have highest quality of evidence. Both antenatal and postnatal PFMT may improve quality of life, reduce urogenital distress and urinary symptoms after delivery [45,46,66,70]. Patients should get at least written instructions how to do PFMT. There is a big variety of questionnaires and methods used to evaluate UI symptoms in RCTs; less than a half of analyzed trials used certified questionnaires. Also, follow-up time after intervention was relatively short. Only a few trials followed patients longer than half a year. More standardized, high sample and longer follow-up studies are needed [66].

Too few trials analyzed PFMT effect on AI and POP prevention. More studies are needed in this field. Late pregnancy is associated not only with higher incidence of UI, but also AI and involuntary loss of flatus. External anal sphincter muscle might be trained the same way as other perineal muscles. Most studies evaluating PFD lack data about involuntary loss of flatus or stool during late pregnancy and postpartum and PFMT effect on this condition. POP reduction in RCT control groups shows that regeneration after delivery improves this condition even without PFMT in a short period (6 weeks to 1 year after delivery), but what is still unclear is whether there a difference between PFMT groups and control groups after a longer time [29,30,31,32,33,34,35,36,43]. Pelvic-floor rehabilitation including various rehabilitation methods and PFMT is recommended as a POP treatment, and there were more severe degrees of POP prevention method in middle-aged women with asymptomatic or mildly symptomatic minor degree POP [73,74]. One of the reasons—that analyzed trials and reviews cannot provide strong evidence of the use of PFMT in POP prevention—might be that there is an increase in intensity of physical activity and weight-lifting in the postpartum period, due to returning to pre-pregnancy lifestyle and additionally baby-care routine which includes baby and baby-stroller lifting. These activities, if performed incorrectly, may increase severity of POP [36,75]. Other possible reason is follow-up period; if patients start training about 6 weeks postpartum and the last follow-up point is 6 months postpartum, the time interval is too short to evaluate the preventative effect of PFMT. There is also a lack of studies with PFMT and perineal rehabilitation timing; according to one trial, there might be a positive effect in POP prevention when PFMT is started very early—second day after delivery—regarding the episiotomy or perineal lacerations [25,26,27,28,29,30,31,32,33,34,35,36,74].

Proposals for research: there is high heterogeneity in RCTs. It is recommended to use more standardized measures—approved questionnaires to evaluate symptoms, use of validated terminology, for example joint IUGA-ICS terminology reports, PFMT reporting, for example use of a Consensus on Exercise reporting template, more attention to antenatal exercises effects, and longer follow-up [15,66]. Still, more evidence is needed about the best timing of PFMT in the postpartum period [25]. There are also some methods that were useful in treating middle-aged women with UI or POP symptoms; however, their effect on prepartum and postpartum women is unknown, for example extracorporeal magnetotherapy [76].

Proposals for practice: prepartum patient counseling about pelvic-floor anatomy and functions and how to prevent PFD during pregnancy and after labor is a necessary point of PFD prevention. Women should be encouraged to perform PFMT in prepartum and postpartum periods, because of the proven positive effect on UI prevention and treatment. National strategies for pregnancy and postpartum PFR programs orientated to PFD prevention should be a priority in national healthcare systems due to the high prevalence of POP and UI and the prediction for them to increase in general female population [2,11,16,66].

## 9. Study Limitations

This study had some limitations: limited number of databases used for literature search; article language was only English; short period of time for article selection; reporting bias due to selective reporting; and exclusion of incomplete articles.

This study did not receive any funding. The authors of the review declare no conflict of interests.

## Figures and Tables

**Table 1:** Clinical trials characteristics.

Theme	Study	Number of Participants	Comparison	Main Findings	Difference Between Groups
SF	(1) Huang L 2019	40	far-infrared radiation (FIR) effectiveness on perineal pain and sexual function (SF) improvement postpartum.	no additional benefit of postpartum FIR on primiparous women undergoing an episiotomy and 2nd degree perineal lacerations.	*p* > 0.05 there was no statistically significant differences between groups.
	(2) Kolberg Tennfjord M 2016	175	PFMT 6 weeks to 6 month postpartum effect on vaginal symptoms or symptoms related to sexual dysfunction. ICIQ-VS, ICIQ-FlUTSsex questionanires were used.	PFMT postpartum may help to reduce symptom: “vagina feels loose or lax”.	*p* = 0.03 symptom: “vagina feels loose or lax”
	(3) Citak N 2010	75	PFMT effect on desire, pain, lubrication, orgasm, female sexual index (FSFI) scores, pelvic-floor muscle strenght 4th and 7th month postpartum.	All domains, except satisfaction, were significantly higher in the training group compared with the controls. Pelvic-floor-muscle strength was found to be increased in the 7th month in the training group.	*p* < 0.001 sexual arousal, lubrication, orgasm, and satisfaction scores were improved in the 7th month in the training group *p* < 0.001 Pelvic-floor-muscle strength was found to be increased in the 7th month in the training group
	(4) Dionisi B 2011	45	intravaginal TENS, myofascial stretching and PFMT effect on postpartum dyspareunia.	Intravaginal TENS and pelvic-floor rehabilitation exercises reduced dyspareunia symptoms and led to an aesthetic improvement on perineal scar.	*p* < 0.05 Dyspareunia symptoms decreased from 2–3 to 0 according to the Marinoff Dyspareunia Scale
	(5) Golmakani N 2015	79	Kegel excersises after delivery effect on pelvic-floor muscle strength and on sexual self-efficacy.	Kegel exercises 8 weeks after delivery for 8 weeks improved pelvic-floor muscle strength and increased sexual self-efficacy scores in the intervention group.	*p* = 0.001 sexual desire, *p* = 0.001 arousal, *p* < 0.0001 orgasm, *p* = 0.001 body acceptance
	(6) Zare Z 2014	79	effect of pelvic-floor muscle exercises on sexual quality of life in primiparous women after childbirth.	8 weeks of PFMT has positive effect on sexual quality of life, marital satisfaction and pelvic-floor muscle strength starting from 8 weeks after childbirth.	*p* < 0.0001 pelvic-floor muscle strength *p* < 0.0001 marital satisfaction *p* < 0.001 sexual satisfactions *p* < 0.001
	(7) Iervolino S A 2017	70	Intensive supervised excersises vs. home excersises 6 months after delivery effect on female sexual dysfunction (FSFI questionnaire).	There were significant improvement for the average scores of all FSFI domains except Pain Domain, while a significant change in the Pain Domain is achieved only in the intensive supervised excerises group.	No *p* values published
POP and/or UI and/or AI	(8) Yang S 2017	189	Direct Vagina Low Voltage Low Frequency Electric Stimulation (DES) Effect on POP, Incontinence severity, pelvic-floor muscle electrophisiology.	There were differences between control group, PFMT group and PFMT plus DES groups 3 months postpartum. Rehabilitation exercises combined with DES effectively reduced maternal pelvic organ prolapse and the extent of maternal urinary incontinence and enhanced pelvic-floor muscle strength.	*p* < 0.0001 POP-Q grade, *p* < 0.0001 the degree of incontinence score, *p* < 0.0001 Oxford score of pelvic-floor muscle strength *p* < 0.0001 sustained contraction pressure of type I muscle fibers, the rapid contraction pressure *p* = 0.001 shrink number (*n*/6s) of the type II muscle fibers.
	(9) Sun Z 2015	324	electrical stimulation and biofeedback treatment effect on pelvic-floor electrical physiological indexes, pelvic-floor dysfunction prevention and quality of life.	Muscle fibers strength and POP-Q scores improved in intervention group after 6 weeks and after 12 month. There were no differences in quality of life (PISQ-12 and PFIQ-7 scores).	*p* < 0.01 Point Aa *p* < 0.01 proportion above level III of type I and type II muscle fibers strength *p* > 0.05 questionnaires in quality of life and quality of sexual life.
	(10) Glazener C M 2014	747	long-term (12-year) effects of a nurse-led PFMT on postnatal UI, AI and POP, compared to control group.	There were no statistically significant differences in any outcomes: POP, UI, AI.	UI ess than once per week at trial entry *p* = 0.673 UI at least once per week at trial entry *p* = 0.284 FI Participants with fecal incontinence at trial entry *p* = 0.987 FI Participants without fecal incontinence at trial entry *p* = 0.183 POP-Q stage at hymen or beyond *p* = 0.883 POP-Q stage ≥ 2 stage *p* = 0.954
	(11) Lekskulchai O 2014	219	Effect of antenatal PFMT on bladder neck descention and bladder symptoms in nulliparous pregnancies vs * control group.	There were no differences between groups in urinary tract symptoms. PFMT may reduce bladder neck mobility at 6 month after childbirth.	control group BND (16.4 ± 6.6 mm) PFMT group BND (13.9 ± 7.3 mm) *p* = 0.03
	(12) Wenjuan L 2020	67	Effect of transvaginal electrical stimulation (TVES) 5 times A group vs. B group: TVES 3 times plus EMG-triggered neuromuscular stimulation 2 times on postpartum woman with extremely weak pelvic muscle strength (pelvic muscle contractility, PFIQ-7, PFDI-20, PISQ-12, IIQ-7 questionnaires)	Muscle contraction were elevated in both groups. No significance difference found between groups. No significant difference of questionnaire had been detected between baseline and after treatment in 2 groups.	Pelvic-floor muscle contraction group A vs. group B *p* = 0.57
	(13) Stafne S N 2012	855	12 weeks excersise programme between 20 and 36 weeks of gestation vs. standart antenatal care effect on UI and AI in late pregnancy.	Differences between groups were not statistically significant, but fewer woman in PFMT group had UI and AI self-reported symptoms.	weekly urinary incontinence (11 vs. 19%, *p* = 0.004) fecal incontinence (3 vs. 5%, *p* = 0.18)
	(14) Rydningen M 2017	58	Woman with AI after obstetrical anal sphincter injury were classified in two groups Permacol injection or sacral neuromodulation. outcomes were: the difference in the St Mark’s incontinence score between baseline and 6 months changes in (FIQL) and (ICIQ-UI-SF) scores.	Sacral neuromodulation was more effective than Permacol injection at all outcome measures.	St Mark’s score *p* < 0.0001 Four scales (lifestyle, coping, depression, embarrassment) of FIQL-disease-specific quality of life questionnaire *p* < 0.001 (lifestyle, coping, embarrassment scales) *p* = 0.005 (depression scale) urinary incontinence (ICIQ-UI-SF) scores *p* = 0.002
	(15) Berman J 2019	50	bipolar vaginal radiofrequency device (VotivaTM, InMode) effect on pelvic-floor muscle tone, pelvic-floor dysfunction and patient perception of improvement index (PSI) in post-partum women.	PSI improvement correlated with number of treatments. This study showed improvement in maximal pelvic-floor contraction after treatment with bipolar vaginal radiofrequency device. No changes were found in resting muscle tone.	No changes were found in resting pelvic muscle tone after Votiva however the number of treatments appeared to impact mean values of maximal pelvic-floor contraction *p* < 0.001.
	(16) Artymuk N 2020	70	PFMT using EmbaGYN (group I) or Magic Kegel Master (group II) devices post-partum effect on PFDI-20, FSFI questionnaires.	After 4 weeks PFMT, there was a significant decrease in the rates of all PFD symptoms including pelvic organ prolapse and urinary and/or fecal incontinence in both groups. The rates of sexual dysfunction decreased significantly only in Group II.	Sexual dysfunction symptoms decreased in group II 69.4 versus 25.0% *p* = 0.001 symptoms of urgent urinary incontinence versus baseline. Group I 35.3 versus 8.8% *p* = 0.009 rates of urine leakage independent of physical activity (Group I) decreased from 23.5 to 5.9% *p* = 0.040
	(17) Glazener C M 2001	747	Control group vs. intervention group: assessment by nurses of UI with conservative advice on PFMT at 5, 7, and 9 months after delivery supplemented with bladder training at 7 and 9 months.	Signifficant improvement on UI in intervention group. Fecal incontinence was also less common in intervention group. Women in intervention group were more likely to still perform PFMT 12 months after delivery.	UI *p* = 0.037 AI *p* = 0.012 Performing excerises 12 month postpartum *p* < 0.001
Episiotomy, obstetrical trauma	(18) Ka Lai Shek 2011	146	Incidence of pelvic-floor injury evaluated with four-dimensional translabial ultrasonography. Intervention group used Epi No trainer from 37 weeks.	No significantly lower incidence of pelvic-floor muscle injury in Epi No group.	Reduction in levator avulsion and microtrauma *p* ≥ 0.22
	(19) Leon-Larios F 2017	466	Intervention group: daily perineal massage and pelvic-floor exercises from 32 weeks of pregnancy Vs standart care.	There was a significant reduction in episiotomy rates in intervention group, also less III-IV degree tears, less postpartum perineal pain.	reduction in episiotomy 50.56% versus 82.19%, *p* < 0.001 higher likelihood of having an intact perineum 17.61% versus 6.85%, *p* < 0.003 third-degree tears 5.18% versus 13.12%, *p* < 0.001 fourth-degree tears 0.52% versus 2.5%, *p* < 0.001 less postpartum perineal pain 24.57% versus 36.30%, *p* < 0.001
	(20) Peirce C 2013	120	Effect of early home biofeedback physiotherapy after third-degree perineal tear.	no added value in using early home biofeedback physiotherapy in the management of women sustaining third-degree tears. There was no significant difference in anal resting and squeeze pressure values and in symptom score and quality of life measurements between the groups.	anal resting and squeeze pressure values *p* = 0.123 and *p* = 0.68
	(21) Dietz HP 2014	660	Effect of Epi No device on perineal trauma prevention. Intervention group used Epi No device from 37 week, all subjects were evaluated by POP-Q assessment and 4D translabial ultrasound.	There were no evidence for a protective effect of the antenatal use of a the Epi No, on pelvic-floor structures in primiparae giving birth to a term singleton after uncomplicated pregnancies.	absolute risk reduction *p* = 0.39 clinical anal sphincter trauma *p* = 0.77 perineal tears *p* = 0.65
	(22) Oakley S H 2016	304	PFMT effect on the quality of life and function in women 12 weeks after OASIS ***.	All women showed improvements in quality of life and function at 12 weeks after delivery, regardless of treatment.	Fecal Incontinence Quality of Life domain scores improved: coping *p* = 0.006 depression *p* = 0.009 anorectal manometry, squeezing pressure improved *p* = 0.035
UI	(23) Sut H K 2016	60	PFMT using a computer-based system effect on pelvic-floor muscle strength, urinary symptoms, quality of life, and voiding functions.	Pelvic-floor muscle exercises applied during pregnancy and the postpartum period increase pelvic-floor muscle strength and prevent deterioration of urinary symptoms and quality of life in pregnancy.	pelvic-floor muscle strength *p* < 0.001 UDI-6, IIQ-7, and OAB-q scores during weeks 36–38 of pregnancy in the Training group *p* > 0.05
	(24) Mørkved S 2003	301	Intensive, supervised PFMT during pregnancy effect on PFMS ** and self-reported symptoms of urinary incontinence.	Less urinary incontinence symptoms and higher PFMS in PFMT group at 36 week of pregnancy and 3 months postpartum.	urinary incontinence at 36 weeks *p* = 0.007 3 months after delivery *p* = 0.018 PFMS at 36 weeks’ pregnancy *p* = 0.008 3 months after delivery *p* = 0.048
	(25) Ahlund S 2013	100	PFMT 10–16 weeks postpartum effect on symptomatic woman with UI.	Maximally voluntary contraction increased and Self-reported symptoms of UI was significantly improved in both groups. Written training instructions were as efficient as home-based training with follow-up visits every sixth week.	Self-reported symptoms of urinary incontinence *p* < 0.05 Maximally voluntary contraction *p* < 0.05
	(26) Kocaöz S 2013	102	PFMT as a prevention of UI in prepartum period and after delivery.	There were statistically significant differences between development of UI in intervention and control groups. In intervention group were less UI 28th and 32nd weeks of gestation and the 12th week postpartum.	UI development in control group 30, 48 and18%; intervention group 5.8, 17.3 and 1.9% *p* < 0.05
	(27) Szumilewicz A 2020	260	PFMT from the 2nd trimester of pregnancy with surface electromyography biofeedback and instructions how to exercise postpartum effect on the UI 2 months and 1 year postpartum.	2 months after birth, for the symptomatic women the Incontinence Impact Questionnaire (IIQ) scores were significantly lower than in PFMT group.	Lower IIQ scores in PFMT group 2 months postpartum *p* = 0.002
	(28) Reilly E T C 2014	268	Supervised PFMT monthly intervals from 20 weeks until delivery effect on primigravidas with increased bladder neck mobility.	Fewer postpartum stress incontinence in PFMT group. There was no change in bladder neck mobility and no difference in PFMS.	postpartum stress incontinence 19.2% PFMT group 32.7% in the control group.
	(29) Sangsawang B 2016	70	Supervised PFMT for 6 weeks prepartum effect on UI.	Fewer women in the intervention group reported UI than in the control group at 38 gestational week.	Self-reported UI 27.3% PFMT group versus 53.3% control group. *p* = 0.018
	(30) Dumoulin C 1995	8	Pelvic-floor neuromuscular electrostimulation combined with PFMT effect on postpartum UI treatment.	Both the quantity of urine loss and the frequency of incontinence were lower following the implementation of the physical therapy program. Five subjects became continent and three others improved.	-
	(31) Dinc A 2009	80	Supervised PFMT during pregnancy effect on UI during pregnancy and postpartum.	PFMT group had a significant decrease in UI 36 to 38 weeks of pregnancy and 6 to 8 weeks postpartum periods, and their PFMS increased to a larger extent. Control group had an increase in the PFMS in the incontinence episodes in the postpartum period.	Episodes of UI 36 to 38 weeks *p* = 0.008 Postpartum 6–8 weeks *p* = 0.014
	(32) Lee I S 2006	49	PFMT after delivery with biofeedback and electrical stimulation effect on PFMS and urinary symptoms.	PFMS increased in intervention group and subjective lower urinary symptoms decreased in this group.	Increase of PFMS in intervention group at the end of treatment *p* = 0.0001 Decrease of urinary symptoms in intervention group: Enuresis *p* = 0.022 UI *p* = 0.038 urge incontinence *p* = 0.041 frequency of incontinence *p* = 0.005 amount of incontinence *p* = 0.003
	(33) Joseane Marques 2012	33	Effect of PFMT over pelvic-floor muscle contractility and UI in pre- and postpartum periods.	PFMS increased after the training program for all groups (primigravid pregnant women, postpartum primiparous, postpartum primiparous women). The scores of both ICIQ-UI SF and ICIQ-OAB decreased.	Increase of PFMS *p* = 0.0001 Decrease of scores: ICIQ-UI SF *p* = 0.009 ICIQ-OAB *p* = 0.0003
	(34) Sangsawang B 2012	66	PFMT effect on on the severity of stress UI in pregnant women.	The 6-week PFMT programme was able to decrease the severity of symptoms in pregnant women with stress UI.	Frequency of UI *p* < 0.001 Perceived UI *p* < 0.001
	(35) Woldringh C 2007	264	PFMT during pregnancy for woman who already have UI effect on UI.	UI decreased strongly after pregnancy, irrespective of usual care or PMFT during pregnancy.	Decrease of the mean score of UI in control and intervention group *p* < 0.001 Difference between groups *p* = 0.329
	(36) Dumoulin Ch 2004	64	Multimodal supervised physiotherapy effect on persistent postpartum UI.	Scores on the pad test, Visual Analog Scale, Urogenital Distress Inventory, and Incontinence Impact Questionnaire improved significantly in both treatment groups, whereas no changes were observed in the control group.	multimodal pelvic-floor rehabilitation group *p* < 0.002 multimodal pelvic-floor rehabilitation with abdominal muscle training group *p* < 0.002
	(37) Mørkved S 2000	162	Long-term effect of a postpartum PFMT course in prevention and treatment of UI.	PFMT course was effective in the prevention and treatment of stress UI. At the 1 year follow-up, significantly more women in control group than in the training group reported stress UI/or showed urinary leakage at the pad test.	Difference between control and intervention group (UI and pad test) *p* < 0.01.
	(38) Pelaez M 2014	169	Effect of PFMT taught in a general exercise class during pregnancy on the prevention of UI in nulliparous continent pregnant women.	PFMT taught in a general exercise class three times per week for at least 22 weeks was statistically significantlly effective in primary prevention of UI in primiparous pregnant women.	Reported frequency of UI *p* < 0.001 Amount of leakage *p* < 0.001 ICIQ-UI SF Score *p* < 0.001
	(39) Wilson p D 1998	230	Effect of PFMT on UI reduction for incontinent woman postpartum.	The prevalence of incontinence was significantly less in the intervention group than in the control group. There were no significant differences between the groups as regards perineometry measurements or pad test results.	The prevalence of incontinence *p* = 0.0003
	(40) Ewings p 2005	723 (recruited and assesed for risk factors) 234 (Included in trial)	Assessment of risk factors for developing UI following childbirth, and effect of physiotherapist-led intervention to reduce incidence of UI.	The intervention as designed did not help in preventing future incontinence. Chronic constipation and episiotomy in at least one delivery were independent risk factors, while an epidural or spinal was protective.	Chronic constipation *p* = 0.04 At least one episiotomy *p* = 0.004 Recent epidural/spinal *p* = 0.02
	(41) Cavalcante de Assis L 2015	87	Effect of illustrated PFMT guide to prevent UI during pregnancy.	Less woman who performed PFMT were incontinent compared to control group. To evaluate continence miction diary was used.	UI frequency *p* < 0.001
	(42) Sampselle C M 1998	46	PFMT effect on symptoms of stress UI and PFMS in primigravidas during pregnancy and postpartum.	Practicing PFMT results in fewer UI symptoms during late pregnancy and postpartum. Diminished UI symptoms were seen in the treatment group, with significant treatment effects demonstrated at 35 weeks gestation and 6 weeks postpartum and 6 months postpartum.	35 weeks gestation *p* = 0.043 6 weeks postpartum *p* = 0.032 6 months postpartum *p* = 0.044
	(43) Dumoulin Ch 2013	57	long-term effect of intensive, 6-week physiotherapy programs, on persistent postpartum stress UI.	Benefits of physiotherapy for postpartum UI is still present 7 years post-treatment. There were no statistically significant differences in Pad test, UDI ant IIQ scores.	Pad test *p* = 0.082 UDI *p* = 0.10 IIQ *p* = 0.05
	(44) Ptak M 2019	137	PFMT combined with abdominal muscle training or just PFMT effects on stress UI after vaginal delivery.	Both the combined training of the PFMT and abdominal muscles and the isolated PFMT improve the QoL of women with stress UI.	Summed ICIQ-LUTSqol scores *p* < 0.001
AI	(45) Johannessen H H 2017	109	PFMT effect on postnatal AI.	There was a significant difference in the reduction of St. Mark’s scores from baseline to postintervention in favor of the PFMT group. No differences in manometry measures of anal sphincter length and strength.	Reduction of St. Mark’s scores *p* = 0.040
POP	(46) Bø K 2015	175	PFMT effect on prevention and treatment of symptoms and signs of POP in primiparous postpartum women.	No effect was found of postpartum PFMT on POP in primiparous women.	POP-Q stage *p* = 0.66

* vs.—versus. ** PFMS—pelvic-floor muscle strength. *** OASIS—obstetric anal sphincter injury. SF—sexual function. POP—pelvic organ prolapse. AI—anal incontinence. UI—urinary incontinence. ICIQ-VS. International consultation on incontinence (ICIQ) modular questionnaire—vaginal symptoms. ICIQ-FLUTSsex.I—CIQ sexual matters module. ICIQ-UI SF—International Consultation on Incontinence Questionnaire-short form. ICIQ-OAB—International Consultation on Incontinence Questionnaire Overactive Bladder. UDI—Urogenital Distress Inventory. IIQ—Incontinence Impact Questionnaire. QoL—quality of live. ICIQ-LUTSqol—International Consultation on Incontinence Questionnaire Lower Urinary Tract Symptoms Quality of Life Module. POP-Q—Pelvic Organ Prolapse Quantification System.

**Table 2:** Systematic reviews characteristics.

Author, Years	Name of the Study	Number of Articles Analyzed	Main Conclusions	Level of Evidence (GRADE)
(1) Schreiner L 2018	Systematic review of pelvic-floor interventions during pregnancy.	22	PMFT during pregnancy shortened the second stage of labor and reduced UI; Perineal massage reduced perineal pain; Use of the Epi No device tended to have no effect.	⨁⨁◯◯ LOW Due to imprecision and selective outcome reporting
(2) Lemos A 2008	Do perineal exercises during pregnancy prevent the development of urinary incontinence? A systematic review.	4	PFMT may be effective at reducing the development of postpartum UI, despite clinical heterogeneity among the RCT.	⨁⨁⨁◯ MODERATE
(3) Woodley S J 2020	Pelvic-floor muscle training for preventing and treating urinary and fecal incontinence in antenatal and postnatal women. Cochrane Systematic Review.	46	Antenatal PFMT probably decreases the risk of UI in late pregnancy. No evidence that PFMT to treat postnatal UI results in a difference in UI in the late postnatal period. A minimum follow-up of six months postnatally is probably more useful to be sure how many cases of UI or AI are persistent. For treatment studies, while a postintervention measure is useful, data on the duration of effect (e.g., one year or longer) are needed. Pregnancy and birth appear to be the most consistent and important factors associated with the development of UI and AI in women.	⨁⨁⨁⨁ HIGH
(4) Wagg A 2007	Unassisted pelvic-floor exercises for postnatal women: a systematic review.	4	unassisted PFMT may be helpful in reducing postnatal incontinence, but that effects may not be maintained over time.	⨁⨁◯◯ LOW Due to imprecision and selective outcome reporting
(5) Sobhgol S S 2019	The Effect of Pelvic-Floor Muscle Exercise on Female Sexual Function During Pregnancy and Postpartum: A Systematic Review.	10	postnatal PFMT was effective in improving Sexual function (SF). However, there is a lack of studies describing the effect of PFMT on SF during pregnancy, and only minimal data are available on the postpartum period.	⨁◯◯◯ VERY LOW Due to high risk of bias and small sample sizes
(6) Hadizadeh-Talasaz Z 2019	Effect of pelvic-floor muscle training on postpartum sexual function and quality of life: A systematic review and meta-analysis of clinical trials.	12	Evidence showed that PFMT in primi or multi-parous women can boost SF in postpartum and it is a safe strategy. The review of these studies has some implications for practice. It has been suggested that postpartum women who do PFMT may benefit from this procedure, increasing sexual health and QoL. Therefore, health professionals should encourage women to do postnatal exercise.	⨁⨁◯◯ LOW Due to high risk of bias and small sample sizes
(7) Mørkved S 2013	Effect of pelvic-floor muscle training during pregnancy and after childbirth on prevention and treatment of urinary incontinence: a systematic review.	22	PFMT is effective when supervised training is conducted. Further high-quality RCTs are needed especially after delivery. Given the prevalence of female UI and its impact on exercise participation, PFMT should be incorporated as a routine part of women’s exercise programmes in general.	⨁⨁⨁◯ MODERATE Due to large heterogeneity
(8) Wu Y M 2018	Pelvic-Floor Muscle Training Versus Watchful Waiting and Pelvic-Floor Disorders in Postpartum Women: A Systematic Review and Meta-analysis.	15	It remains uncertain whether postpartum PFMT improves POP symptoms because of very low-quality evidence. The POP staging will likely not change with postpartum PFMT. The PFMT may result in improved postpartum SF compared to watchful waiting, and may provide benefit for AI in women with anal sphincter injuries. Postpartum PFMT likely reduces the risk of UI, particularly stress UI symptoms. There is currently little evidence about postpartum PFMT and long-term pelvic-floor function.	⨁◯◯◯ VERY LOW Due to high risk of bias and indirectness of evidence
(9) Driusso *p* 2020	Are there differences in short-term pelvic-floor muscle function after cesarean section or vaginal delivery in primiparous women? A systematic review with meta-analysis.	11	No difference in short-term PFMS after childbirth between primiparous women who underwent cesarean section or vaginal delivery. Reduced PFMS were identified in women who underwent an episiotomy or instrumented vaginal delivery. Future primary studies with longitudinal designs and long-term follow-up periods are needed to strengthen the quality of evidence and provide more conclusive evidence to guide clinical practice.	⨁◯◯◯ VERY LOW Due to high risk of bias and indirectness of evidence

RCT—randomized clinical trial. QoL—quality of life.

## Data Availability

Not applicable.
